# Improving mental health services for homeless youth in downtown Montreal, Canada: Partnership between a local network and ACCESS Esprits ouverts (Open Minds), a National Services Transformation Research Initiative

**DOI:** 10.1111/eip.12814

**Published:** 2019-06-27

**Authors:** Amal Abdel‐Baki, Diane Aubin, Raphaël Morisseau‐Guillot, Shalini Lal, Marie‐Ève Dupont, Pasquale Bauco, Jai L. Shah, Ridha Joober, Patricia Boksa, Ashok Malla, Srividya N. Iyer

**Affiliations:** ^1^ Department of Psychiatry, Faculty of Medicine University of Montreal Montreal Quebec Canada; ^2^ Centre de recherche du Centre hospitalier de l'Université de Montreal (CRCHUM) Montreal Quebec Canada; ^3^ ACCESS Open Minds Réseau d’intervention de proximité pour les jeunes de la rue (RIPAJ) Montreal Quebec Canada; ^4^ ACCESS Open Minds (Pan‐Canadian Youth Mental Health Services Research Network) Douglas Mental Health University Institute Montreal Quebec Canada; ^5^ *Dans la rue*, a Bilingual Charitable Youth Multi‐Services Organization Serving Homeless Youth Montreal Quebec Canada; ^6^ School of Rehabilitation, Faculty of Medicine Université de Montreal Montreal Quebec Canada; ^7^ Centre de recherche du Centre hospitalier de l'Université de Montréal (CRCHUM) Montreal Quebec Canada; ^8^ Prevention and Early Intervention Program for Psychosis (PEPP) Douglas Mental Health University Institute Montreal Quebec Canada; ^9^ Department of Psychiatry McGill University Montreal Quebec Canada

**Keywords:** community mental health services, early identification, homeless youth, mental illness, service organization, youth mental health, Canada

## Abstract

**Aim:**

In many parts of the world, there is growing concern about youth homelessness. Homeless youth are particularly vulnerable to psychological distress, substance use and mental disorders, and premature mortality caused by suicide and drug overdose. However, their access to and use of mental health care is very limited.

**Methods:**

The *Réseau d'intervention de proximité auprès des jeunes* (RIPAJ), a Montreal network of over 20 community stakeholders providing a wide array of cohesive services, was created to ease homeless youth's access to mental health and psychosocial services. Its philosophy is that there should be no “wrong door” or “wrong timing” for youth seeking help. In 2014, the network partnered with the pan‐Canadian transformational research initiative, ACCESS *Esprits ouverts*.

**Results:**

Created through this partnership, ACCESS *Esprits ouverts* RIPAJ has been promoting early identification through outreach activities targeting homeless youth and agencies that serve them. An ACCESS Clinician was hired to promote and rapidly respond to help‐seeking and referrals. By strengthening connections within RIPAJ and using system navigation, the site is working to facilitate youth's access to timely appropriate care and eliminate age‐based transitions between services. A notable feature of our program, that is not usually evident in homelessness services, has been the engagement of the youth in service planning and design and the encouragement of contact with families and/or friends.

**Conclusion:**

Challenges remain including eliminating any remaining age‐related transitions of care between adolescent and adult services; and the sustainability of services transformation and network coordination. Nonetheless, this program serves as an example of an innovative, much‐needed, community‐oriented model for improving access to mental health care for homeless youth.

## 1 CONTEXT

Montreal, Canada's second most populous city (Statistics Canada, 2017), is in the country's only province whose sole official language is French. Montreal is also home to many English speakers; other linguistic minorities and immigrants. According to the 2016 census, 15.3% of Montreal's population was classified as low‐income (Statistics Canada, 2017).

Since 1980, there has been a rapid increase in homelessness in Canada, including among youth (Gaetz, Dej, Richter, & Redman, 2016). Youth homelessness is not an exclusively Canadian concern. In the United States, for instance, a recent report estimated that 3.48 million young adults (aged 18 to 25 years) experienced homelessness or precarious housing over a 12‐month period (Morton et al., 2018). In the United Kingdom, it was estimated that 83 000 youth had availed homelessness services for a one‐year period (Clark, Burgess, Morris, & Udagawa, 2015).

In Montreal, a 2015 count estimated that 3016 people (19% under 30 years) were homeless (Latimer, McGregor, Méthot, & Smith, 2015). Notably, this estimate did not include youth living in unstable, precarious, or unsafe housing (e.g., short‐term homelessness; couch‐surfing or squatting in abandoned, unsafe buildings). Further, Indigenous peoples, transgender persons and newcomer immigrants are overrepresented among homeless youth.

Definitions of *youth homelessness* vary across contexts making comparisons difficult. The Canadian Observatory on Homelessness defines the youth homeless as including those “who are living independently of parents and/or caregivers, but do not have the means or ability to acquire a stable, safe or consistent residence” (Gaetz, Gulliver, & Richter, 2014).

Homelessness is both a consequence of and a contributing factor to mental health problems among youth (Folsom et al., 2005; Martijn & Sharpe, 2006). Most mental disorders have their onset in youth and homelessness significantly exacerbates risk (Kessler et al., 2005). Over 85% of homeless youth report high levels of psychological distress (Gaetz, O'Grady, Kidd, & Schwan, 2016). Compared to the general youth population, there is a substantially higher prevalence among homeless youth of mental disorders (Whitbeck, Johnson, Hoyt, & Cauce, 2004) and of specific disorders like psychosis or substance misuse (Martin, Lampinen, & McGhee, 2006; Roy, Haley, & Leclerc, 2004). Self‐harm, and suicidal ideation and behaviours are also more frequent, with completed suicide and drug overdose being leading causes of premature death in this population (Roy, Haley, & Leclerc, 2004). Importantly, rates of mental disorders and distress are higher in youth who have been homeless for longer (Solorio, Milburn, Andersen, Trifskin, & Rodriguez, 2006).

Yet, homeless youth have poor access to and utilization of mental healthcare (Kidd, Slesnick, Frederick, Karabanow, & Gaetz, 2018; Kort‐Butler & Tyler, 2012; Muir‐Cochrane, Fereday, Jureidini, Drummond, & Darbyshire, 2006). In a Canadian national survey, 84% of homeless youth with mental illnesses reported needing services in addition to any they had received (National Learning Community on Youth Homelessness, 2012). There is an urgent need for integrated service models that effectively address the mental health, general health and social support needs of homeless youth; and serve to prevent future homelessness. To this end, a group of community organizations and healthcare institutions that serve homeless youth in downtown Montreal came together in the 2000s.

## 2 THE CREATION OF *RÉSEAU D'INTERVENTION DE PROXIMITÉ AUPRÈS DES JEUNES*

*Réseau d'intervention de proximité auprès des jeunes* (RIPAJ) was initiated by three psychologists from different organizations who, in 2003, held “proximity meetings” to support each other and exchange ideas about how to best provide care for homeless youth (Aubin et al., 2011). The initial participants then started to invite local stakeholders who joined the meetings, eventually creating a network of partners caring for homeless youth. Over 15 years, the group expanded to include about 20 community organizations and institutional stakeholders (see Table 1) to build a network aimed at improving and accelerating access to and continuity of mental healthcare and related services (Abdel‐Baki et al., 2018). Network members include day centres, shelters, housing resources, medical and psychiatric institutions and specialized services such as a supervised injection site. The philosophy behind RIPAJ has been that there is no “wrong door” or “bad timing” for seeking help. A youth can access the services of the network through any RIPAJ partner.

**Table 1:** Primary partners within ACCESS EO RIPAJ and key services that they offer to homeless youth

Community organizations			Housing services			Institutional partners		
Name	Target population	Services	Name	Target population	Services	Name	Target population	Services
*Dans la rue*	12‐25 y old (transition past 25), homeless youth.	Night shelter, supervised flats and day centre offering psychological services, nursery, social services, Emmett‐Johns School, employment programs, veterinarian clinic, clothing, meals and showers.	*Refuge des Jeunes*	17‐25 y old homeless men	Temporary shelter, dormitory, meals and food banks, personal hygiene products and clothing, first aid services, counselling, supervised flats.	*Clinique JAP—ÉQIIP SOL*	16‐30 y old with first psychotic episode.	Social workers, psychiatrists, occupational therapists and nurses.
*Diogène*	18 y old and older with severe mental health issues, SUD, legal issues and/or homelessness.	Outreach, accompaniment and support.	*Passages*	18‐30 y old women in precarious situations	Temporary housing, housing support and social reinsertion.	*Clinique des Jeunes de la Rue*	14‐25 y old, homeless youth.	Nursing, medical and dental care, psychology and social work, psychiatrist, peer helper, accompaniment.
*Cactus*	Anyone.	Prevention of STIs, supervised injection site.	*Maison St‐Dominique*	18 y and older with mental health issues.	Supervised apartments and psychosocial support.	*Centre de Réadaptation en Dépendance de Montréal*	<25 y old with addiction problems.	Addiction rehabilitation services, educational and legal support.
*Groupe d'intervention alternative par les pairs (GIAP)*	Youth in precarious situations 12‐30 y old.	Peer intervention group, STIs prevention, harm reduction interventions.	*Le Tournant*	18‐29 y old homeless men.	Affordable housing, accompaniment and post‐housing support.	*Direction de la protection de la jeunesse*	<18 y old with compromised security and development.	Medical care for all needs, mental health care, housing and legal support.
*Spectre de Rue*	18 y and older.	Prevention of STIs, supervised injection site, street messengers.	*La Maison Tangente*	18‐25 y old homeless youth.	Transitory housing, accompaniment and support.			
*Premier Arrêt—YMCA*	Anyone.	Outreach, psychosocial services.	*Le Foyer des Jeunes Travailleurs et Travailleuses de Montréal*	17‐24 y old youth in precarious situations.	Housing, accompaniment.			
*Médecins du monde*	18 y and older.	Psychosocial support, mobile health clinic.	*En Marge 12‐17*	Youth 12‐17 y old and their parents.	Emergency shelter, short‐term housing, supervised apartments, support for parents.			
			*Dîners St‐Louis*	18‐30 y old.	Transitional housing, day centre, various social services, clothing and meals.			

Abbreviations: STIs, sexually transmitted infections; SUD, substance use disorder.

## 3 MERGING WITH ACCESS ESPRITS OUVERTS: STRONGER STRUCTURE AND SERVICES

To further improve access to and quality of its mental health services, RIPAJ partnered with the national youth mental health services research project, ACCESS *Esprits ouverts* (ACCESS EO), to create ACCESS *Esprits ouverts* RIPAJ (ACCESS EO RIPAJ) in 2014. The five key objectives of ACCESS EO are early identification; rapid access to an initial evaluation; provision of appropriate care within 30 days; continuity of care beyond the age of 18 years and youth and family engagement (Malla et al., 2018).

ACCESS EO RIPAJ has a core team of two leaders, one from an urban academic hospital; and the other from a community organization that runs a day centre, a night shelter and supervised apartments for homeless youth. Three staff members were hired for this project, including a coordinator of all activities and communication within RIPAJ and with outside agencies; an ACCESS Clinician (currently a social worker) to respond to mental health referrals or help‐seeking within 72 hours; and a research assistant to conduct evaluations with youth per the ACCESS EO protocol.

Among the organizations visited most by Montreal homeless youth, the *Dans la rue* day centre was the first within the RIPAJ network to offer integrated services—meals, clothing, psychological and psychosocial services, music therapy, employment support, and alternative integrated schooling—in a youth‐friendly environment. It is a key node within and a major philosophical influence on ACCESS EO RIPAJ. Annually, RIPAJ serves more than 1000 youth of whom about 300 to 350 are new.

## 4 COMMUNITY MAPPING

An initial step upon joining ACCESS EO was to engage in community mapping to understand where and how homeless youth access mental healthcare, the factors hindering and facilitating such access, and youth's experiences and perceptions of accessing and receiving mental healthcare. ACCESS EO RIPAJ convened individual meetings with all partner organizations to complete questionnaires describing their targeted clientele, the services they offered, and their opinion on accessibility to their services and others in the network. Simultaneously, youth were involved in community mapping through different activities; for example, a mental health fair at which they pinpointed the organizations that helped them with their mental health on an inner‐city map of Montreal. This was done in an accessible manner using “post‐it” notes on which they could write and draw. Youth were also invited to comment on the services received within the network. Viewing community mapping as a dynamic exercise that bears repetition, youth are still invited to mark and comment on the same map, which is hanging on a wall of the ACCESS EO RIPAJ youth space at *Dans la rue*. Additional mapping activities have been undertaken in an ongoing graduate project that uses arts‐based qualitative methods like PhotoVoice.

## 5 EARLY IDENTIFICATION

ACCESS EO RIPAJ, with its community and institutional partners spread throughout downtown Montreal, facilitates access to services from several entry points, be they shelters, supervised housing, employment services or general practitioners. The vastness of the network and its broad service scope increase the chances of serving youth with different backgrounds and needs (e.g., acute psychosocial crises, psychiatric disorders, housing‐related distress, or employment‐ or education‐related challenges) at different times (day or night). For many homeless youth, regular pathways cannot facilitate access to care (e.g., needing a referral from a family physician but not having one or being denied a service for not having an address or identity card). Homeless youth may be likelier to be identified as needing appropriate mental healthcare by an integrated service network like ours than when navigating the system alone.

To increase early identification, outreach activities are conducted several times a week in various partner organizations. The ACCESS Clinician is regularly present at the *Dans la rue* day centre to liaise with staff, discuss which youth might need mental healthcare and provide guidance on how to approach youth to discuss their mental health. If needed, the ACCESS Clinician can immediately engage with youth, evaluate their needs and direct or accompany them to appropriate services. Different field workers (e.g., peer support workers, street outreach workers)—and not only healthcare professionals—can provide referrals to promote early identification. Field workers are offered early identification and basic intervention training through planned on‐site training, conferences, monthly meetings of ACCESS EO RIPAJ partners, etc.

Youth‐friendly early identification activities are regularly offered to youth to enhance their mental health literacy, reduce stigma and promote help‐seeking and wellness. Such activities include mental health information sessions, yoga and art sessions, do‐it‐yourself mental health workshops, drum circles, films/film‐making, outdoor activities, adventure therapy and LGBTQ‐themed meetings. Traditional (e.g., news) and social media have also been used to reduce stigma and promote help‐seeking.

To broaden its offer, ACCESS EO RIPAJ has worked with partners to optimize services (e.g., to offer beds/rooms in a transgender‐friendly shelter). Each new partnership/service increases the pool of new potential youth directed to ACCESS EO RIPAJ.

Nonetheless, early identification poses challenges. Some of the most vulnerable youth, often suffering from multiple traumas that hinder trust (Berry, Barrowclough, & Wearden, 2007), may simply not come into contact with RIPAJ organizations. ACCESS EO RIPAJ staff cannot systematically engage with all youth using its services, thus potentially missing some youth in need. In general, many youth can be uncomfortable engaging with personnel because of suspiciousness, active psychiatric symptoms or fears of being misunderstood or stigmatized. Among the homeless, such inhibitors are more pronounced because homeless youth are likelier to have past negative help‐seeking experiences and traumas. Such apprehension may be further compounded for those belonging to minority groups. To enhance youth's comfort in engaging with ACCESS EO RIPAJ, several efforts are underway, including reassuring them of confidentiality and respect for autonomy. While important for all clinical practice, these two values have been highlighted as especially salient by homeless youth and have been part of RIPAJ's practice since its inception in 2003.

Some youth with more severe psychiatric symptomatology and/or with difficulty establishing trust may not be voluntarily amenable to assessment, sometimes necessitating legal means like court‐ordered psychiatric evaluations. The presence of ACCESS EO RIPAJ members at the partner site hospital and links with other emergency services have helped the early identification of youth with severe mental health and substance use. This will likely reduce traumatic pathways to care such as emergency services, court‐ordered psychiatric evaluations and police involvement.

## 6 RAPID, ENGAGING ACCESS

ACCESS EO RIPAJ provides a mental health evaluation within 72 hours after referral/help‐seeking through various ways. The chief strategy has been to hire and train (with support from ACCESS EO central office) a social worker in the ACCESS Clinician role. This clinician can be contacted by telephone, email or in person, and at different sites. Youth can initiate contact by themselves or via a partner organization, for example, through staff at a shelter who often accompany the youth to a first meeting. The first meeting can take place at one of many partner sites, typically one that is already attended by the youth and therefore familiar to them. The ACCESS Clinician frequently visits partner sites, and goes when called, generally ensuring same‐day responses to referrals.

Despite a well‐planned rapid‐access system, some situations make meeting youth within 72 hours challenging. It can be difficult to reach some youth after the initial request if they are inaccessible by phone/e‐mail or are preoccupied with basic needs (e.g., food or shelter). Their nomadic circumstances and ambivalence towards mental health professionals, often because of negative past experiences, can impede engagement in the initial evaluation. Many RIPAJ partners make special efforts to facilitate communication, by offering Internet access, taking messages for youth and having a message board. Nevertheless, it remains difficult to keep in touch with youth who have no means of being contacted, especially if they forbid communication about them between partner organizations.

## 7 APPROPRIATE CARE

ACCESS EO RIPAJ aims to deliver prompt, meaningful medical and psychosocial interventions to youth with mental health problems. Various interventions are available to youth including psychology and psychiatry consultations, psychotherapy, substance use treatment, and legal counselling, typically well within 30 days. These interventions are provided at RIPAJ sites by network professionals or by contracted professionals (e.g., a psychologist from a publicly‐funded institute provides substance‐related interventions at RIPAJ organizations).

A major strength of ACCESS EO RIPAJ is its ability to integrate under one umbrella services that address a range of homeless youth's needs (see Table 1). These include medical, psychological, psychiatric, education, employment, legal, housing, and financial services. For instance, la *Clinique des jeunes de la rue*, created within the province's public primary healthcare system, provides general health services. These include access to general practitioners, dentists, social workers, a psychologist, a psychiatrist and primary care nurses (for mental healthcare and other problems such as sexually transmitted infections, etc.). Psychotherapy and other mental health services are provided at multiple locations like *Dans la rue* and *Clinique des jeunes de la rue* for as long as needed until 25 years old. Additionally, the *ÉQIIP SOL* clinic provides three to five years of specialized services for homeless youth with psychosis, including psychiatry, intensive case management, occupational therapy, housing support, employment and education support and substance use treatment. Multiple psychosocial and educational services are also offered at *Dans la rue*. Depending on their needs, youth can be referred to partner organizations, including housing service providers like *Passages* that offers temporary housing to young homeless women.

All services are free and can often be obtained within 30 days. The ACCESS Clinician works with RIPAJ partners to ensure seamless transitions and exchange of information, and to adapt interventions to the changing needs of youth.

Nonetheless, obstacles remain that impede timely service provision, including difficulty reaching youth or maintaining contact. One way this is being addressed is by the ACCESS Clinician regularly spending time at partner sites, especially *Dans la rue*, to establish direct contact with current service recipients. Within ACCESS EO RIPAJ, doctors volunteer their time to serve those youth who do not have public or private health insurance. The lack of health insurance (whether because of ineligibility or lack of proof) occasionally delays access to services outside the network.

Youth are referred outside RIPAJ for certain specialist services (e.g., for autism, serious eating or personality disorders). In such instances, the referral process may not be as seamless as it is within RIPAJ. External services often require traditional written consultations, have long waiting lists, are poorly integrated and may tend to marginalize and stigmatize homeless youth. For example, some specialized services outside RIPAJ exclude those with comorbid substance use and severe mental illness, compromising access for some of the most vulnerable youth.

## 8 YOUTH AND FAMILY ENGAGEMENT

The most salient change to RIPAJ since joining ACCESS EO has been the involvement of youth and families in the planning and administration processes of partner organizations. Earlier, youth had a limited role in most partner organizations. Some organizations had peer support workers who accompanied youth to care, but few engaged youth in additional ways. In ACCESS EO RIPAJ, youth and family members/carers are invited to join committees, provide input in planning services and activities, and help tailor clinical programs and research to their needs. Youth also act as “ambassadors” and help engage other youth in services, research and stigma‐reduction activities through testimonials, artistic performances, etc. Following an ACCESS EO national youth council recommendation, youth have helped interview potential ACCESS EO RIPAJ staff to evaluate their congruence with the network's values and signalling to them the primacy accorded to the youth voice. A RIPAJ youth sits on ACCESS EO's national youth council, to which she brings the under‐represented perspectives of Francophone and homeless youth. At RIPAJ's partner hospital, youth have prompted significant changes in an inpatient ward's rules to make it more youth‐friendly. These have included extending visiting hours; providing access to Wi‐Fi, electronic devices and art materials; permitting the wearing of youth's own clothes upon admission; allowing direct inpatient admission without having to pass through the emergency room; etc. Overall, youth who have been variously engaged have reported feeling empowered and more satisfied with services.

Although often isolated and often despite negative past traumatic experiences involving family members (Winland, Gaetz, & Patton, 2011), homeless youth can create and preserve positive links with family members and other attachment figures. As family and social support positively impacts service use (Kozloff et al., 2013), RIPAJ personnel help youth resolve difficulties with their relatives and re‐establish family links, *when appropriate*. These links may have been lost because of their mental illness and substance misuse or difficult past experiences. Furthermore, adopting a broad definition of social support, staff also support youth in creating and maintaining other meaningful relationships such as with friends or other significant attachment figures (e.g., peer mentor).

Nevertheless, engaging youth and especially families/carers and sustaining their involvement can be challenging. This is attributable to the marginalized status of homeless youth, their often‐complicated relations with family, and their families' own challenges. Moreover, youth living in precarious situations spend a lot of time addressing basic needs, and find it difficult to stay involved over longer periods of time.

There is wide variation in the spectrum of youth engagement across RIPAJ organizations with some actively partnering with youth in service planning and delivery, and others engaging youth only as collaborative service recipients. Also, not all RIPAJ organizations have the requisite training to engage families/carers. While some services have been adapted in response to youth and family recommendations, this has proven harder in institutional partner settings. Sustained, creative efforts are still needed to ensure the meaningful involvement of marginalized youth and families.

## 9 ELIMINATING AGE‐BASED TRANSITIONS

Like in many parts of the world, those receiving care in Quebec's children's mental health services are moved to the adult healthcare system at the age of 18 years. Also, children in the youth protection system (in other jurisdictions, referred to as looked‐after children or children in care) are transitioned out at the age of 18. When these transitions are not well organized, which is often the case for homeless youth, it can result in delays or interruptions in care provision as some services are neither available in the adult system nor developmentally suitable. The situation is even worse for youth who received little or no mental healthcare before adulthood.

To ensure the continuity of care into adulthood (if needed), various RIPAJ partners have eliminated age‐based transitions, instead articulating inclusion criteria around needs and offering services from early or mid‐adolescence up to 25 to 30 years. When age‐based transitions become unavoidable, RIPAJ workers plan transfers well in advance and accompany youth as they connect to appropriate adult services.

Over a 10‐year course of collaboration, RIPAJ encouraged youth protection services to establish two single‐sex group homes for 16‐ to 21‐year‐olds with severe mental illness. Staff at these supervised homes work with treating teams and are being trained to be more familiar with severe mental illnesses and youth needs.

Despite these efforts, easing youth's transitions into adulthood remains arduous. ACCESS EO RIPAJ has limited influence on the organization of care in external services. Consequently, some youth continue experiencing abrupt, age‐based discontinuities of care. By demonstrating the effectiveness of its approach, ACCESS EO RIPAJ hopes to positively influence policy in this regard.

## 10 VIGNETTE

To illustrate the functioning of ACCESS EO RIPAJ, we present an anonymized vignette of a youth's journey through services. Originally from the countryside, “C” had been placed in youth protection as a preadolescent after his mother died of cancer. When he turned 18 and left foster care, he dropped out of school and moved in with acquaintances. His drug intake increased and he became increasingly disorganized. When conflicts erupted, C was evicted from the apartment. He was repeatedly brought by the police to emergency rooms in states of acute intoxication and despite multiple assessments ending in referrals to mental health services, C faced a long waiting list to see a psychologist and did not engage in the proposed psychiatric follow‐up. Eventually, he moved to Montreal and began frequenting shelters to eat and sleep. C confided to a RIPAJ shelter worker whom he had known for a few weeks and come to trust, that he had begun experiencing psychotic symptoms. With his permission, the shelter worker contacted the ACCESS Clinician. C was met the same day at the shelter for an evaluation and the ACCESS Clinician arranged an appointment with a psychiatrist a few days later.

The shelter worker in whom he had initially confided was present for this appointment, helping C develop trust in his mental healthcare team. C's psychiatric symptoms were stabilized with the help of the early intervention for psychosis team, while he lived in a RIPAJ group home until he developed more autonomy. With his health and housing thus stabilized, C expressed interest in returning to school, and registered at the *Dans la rue* school. Within a year, he moved into a supervised apartment run by a partner organization and started working. Before RIPAJ became an ACCESS EO site, a youth like C would not have received an evaluation on the same day and would not have been supported until and nor accompanied to his appointment with the psychiatrist, by the RIPAJ network workers. While the various organizations involved in C's care prior to ACCESS EO RIPAJ were linked, their ability to work collaboratively has been greatly enhanced because of the ACCESS Clinician's role, the regular inter‐organization meetings that now occur and the coordination provided by a dedicated staff member hired for the project.

## 11 DISCUSSION AND CONCLUSION

RIPAJ joined the ACCESS EO network with strong links between various community and institutional stakeholders who were aligned around the common aim of simplifying and expediting access to mental healthcare for homeless youth. Since joining ACCESS EO, RIPAJ has been further strengthened by hiring a coordinator and an ACCESS Clinician who help youth navigate the existing system; by increased youth, community and families' involvement and by enhanced data collection to help measure the impact of the ACCESS EO RIPAJ intervention on youth outcomes and experiences. Through ACCESS EO RIPAJ, network partners have strengthened their commitment to better meeting the mental health needs of homeless youth in Montreal including the aim of eliminating strictly age‐based transitions. The involvement in RIPAJ of some organizations (and, thereby, the continued serving of related needs) remains dependent on clinicians volunteering time. Sustaining the efforts, initiatives and philosophies of ACCESS EO RIPAJ will require service administrators and policy‐makers to embrace the ACCESS EO RIPAJ transformation and their roles therein.

Efforts are underway to ensure the sustenance of the RIPAJ partnerships beyond the duration of the ACCESS EO project. We are documenting the transformation process and key factors contributing to improved access and outcomes by involving youth in data collection. Regular stakeholder meetings are being held to help integrate ACCESS EO values into all RIPAJ youth services. The diversity of our partners, though a core strength, makes the harmonization of practices difficult. A sustainability‐related priority that we have identified is ensuring the retention of the positions of the project coordinator and the ACCESS Clinician. The coordinator will ensure the maintenance of smooth links between network partners and the continuation of common training and early identification activities. The ACCESS Clinician will ensure continued early identification, rapid access and navigation supports. We will also strive to sustain the elimination of age‐based transitions. For unavoidable transitions, we will continue easing the process by accompanying youth.

Our model serves as an example for other initiatives that seek to address the mental health needs of homeless youth. As our experience has shown, homeless youth are well‐served by an integrated services approach in which mental healthcare is part of a broad‐spectrum compendium of services and supports. Facilitating service access for hard‐to‐reach youth also requires mobile clinicians who are flexible enough to meet youth where and when they desire. The structure of ACCESS EO RIPAJ itself represents an innovative approach to cross‐sectoral and inter‐services integration whereby diverse services align around a common objective without losing their individual identities and strengths or requiring co‐location or extensive restructuring.
